# Large-scale lipidomic profiling identifies novel potential biomarkers for prion diseases and highlights lipid raft-related pathways

**DOI:** 10.1186/s13567-021-00975-1

**Published:** 2021-07-21

**Authors:** Yong-Chan Kim, Junbeom Lee, Dae-Weon Lee, Byung-Hoon Jeong

**Affiliations:** 1 Korea Zoonosis Research Institute, Jeonbuk National University, Iksan, Jeonbuk 54531 Republic of Korea; 2 Department of Bioactive Material Sciences and Institute for Molecular Biology and Genetics, Jeonbuk National University, Jeonju, Jeonbuk 54896 Republic of Korea; 3 Metabolomics Research Center for Functional Materials, Kyungsung University, Busan, 48434 Republic of Korea; 4 Department of Bio-Safety, Kyungsung University, Busan, 48434 Republic of Korea

**Keywords:** Prion, Lipid, Lipidomics, Biomarker, Pathway, Lipid raft, GPI, LIPEA

## Abstract

Prion diseases are transmissible spongiform encephalopathies induced by the abnormally-folded prion protein (PrP^Sc^), which is derived from the normal prion protein (PrP^C^). Previous studies have reported that lipid rafts play a pivotal role in the conversion of PrP^C^ into PrP^Sc^, and several therapeutic strategies targeting lipids have led to prolonged survival times in prion diseases. In addition, phosphatidylethanolamine, a glycerophospholipid member, accelerated prion disease progression. Although several studies have shown that prion diseases are significantly associated with lipids, lipidomic analyses of prion diseases have not been reported thus far. We intraperitoneally injected phosphate-buffered saline (PBS) or ME7 mouse prions into mice and sacrificed them at different time points (3 and 7 months) post-injection. To detect PrP^Sc^ in the mouse brain, we carried out western blotting analysis of the left hemisphere of the brain. To identify potential novel lipid biomarkers, we performed lipid extraction on the right hemisphere of the brain and liquid chromatography mass spectrometry (LC/MS) to analyze the lipidomic profiling between non-infected mice and prion-infected mice. Finally, we analyzed the altered lipid-related pathways by a lipid pathway enrichment analysis (LIPEA). We identified a total of 43 and 75 novel potential biomarkers at 3 and 7 months in prion-infected mice compared to non-infected mice, respectively. Among these novel potential biomarkers, approximately 75% of total lipids are glycerophospholipids. In addition, altered lipids between the non-infected and prion-infected mice were related to sphingolipid, glycerophospholipid and glycosylphosphatidylinositol (GPI)-anchor-related pathways. In the present study, we found novel potential biomarkers and therapeutic targets of prion disease. To the best of our knowledge, this study reports the first large-scale lipidomic profiling in prion diseases.

**Supplementary Information:**

The online version contains supplementary material available at 10.1186/s13567-021-00975-1.

## Introduction

Prion diseases are fatal and irreversible neurodegenerative disorders caused by the abnormally folded prion protein (PrP^Sc^), which originates from the normal prion protein (PrP^C^) [1–6]. Under normal physiological conditions, PrP^C^ is located in lipid rafts via glycosylphosphatidylinositol (GPI)-anchoring and plays essential roles in signal transduction, neuroprotection and metal ion homeostasis [7–9]. However, under pathological conditions, PrP^C^ is converted into PrP^Sc^. PrP^C^ conversion may induce a loss of innate physiological functions but also confers deleterious effects in the host CNS and induces spongiform vacuolation and astrocytosis [10–12].

Previous studies have reported that the lipid raft, which consists of glycosphingolipids, cholesterol and protein receptors organized in lipid microdomains, is critically associated with the conformational conversion process of PrP^C^ into PrP^Sc^ [13, 14]. An in vitro prion disease model showed impaired cholesterol metabolism, and the reduction in cellular cholesterol levels by lovastatin, filipin and squalestatin contributed to diminishing PrP^Sc^ formation [15–18]. In addition, mislocalization of PrP^C^ by replacement or removal of the GPI anchor inhibited the conversion of PrP^C^ into PrP^Sc^ [19, 20]. Strikingly, a recent study reported that phosphatidylethanolamine acts as an independent cofactor for in vitro PrP^Sc^ formation in the absence of genetic materials [21]. Although several studies have suggested that lipids are significantly related to the conversion process of PrP^Sc^, lipidomic profiling has not been reported in prion diseases thus far.

In the present study, we performed a lipidomic analysis in a mouse model of prion disease and identified novel lipid biomarkers of prion disease and altered lipid-related pathways. For this, wild-type mice were injected intraperitoneally with phosphate-buffered saline (PBS) and mouse-adapted ME7 scrapie prions and sacrificed at different time points (3 and 7 months) post-injection. In addition, we carried out western blotting analysis to detect PrP^Sc^ in prion-infected mice. Furthermore, we performed liquid chromatography mass spectrometry (LC/MS) and analyzed the lipidomic profiling between non-infected mice and prion-infected mice to identify novel lipid biomarkers for prion disease. Finally, we analyzed the altered lipid-related pathways by a lipid pathway enrichment analysis (LIPEA).

## Materials and methods

### Ethical statements

The mouse ME7 scrapie strain was obtained from The Roslin Institute, The University of Edinburgh. C57BL/6J mice were purchased from Nara Biotech (Pyeongtaek, Korea). All efforts were made to minimize the number of animals used and their suffering. All experimental procedures were accredited by the Institute of Animal Care and Use Committee of Jeonbuk National University (JBNU 2020-080).

### Inoculation of mice to investigate lipidomic profile

Six C57BL/6 mice (6-week old) were inoculated by intraperitoneal injection with 100 μL of 1% (w/v) brain homogenate prepared from terminally ill ME7-infected mice. Post-inoculation, the mice were sacrificed at 3 (*n* = 3) and 7 months (*n* = 3). As control, six C57BL/6 mice (6-week old) were inoculated via the intraperitoneal route with 100 μL of PBS. Post-inoculation, the mice were sacrificed at 3 (*n* = 3) and 7 months (*n* = 3). Prion disease symptoms including ataxia, tail rigidity and kyphosis were observed in prion-infected mice at 7 months post- injection. The incubation period of ME7-infected mice was 227 ± 1 days (*n* = 3).

### Western blotting analysis to detect PrP^Sc^

The brains were extracted and homogenized with 10% volumes of RIPA lysis buffer (Thermo Fisher Scientific, USA) containing a cocktail of protease inhibitors (Roche, Germany). To detect specifically PrP^Sc^ in the mouse brain, 40 µg/mL proteinase K was added for 1 h at 37 °C. The samples were heated to 95 °C for 10 min before being loaded into each lane in a 12% sodium dodecyl sulfate (SDS)-polyacrylamide gel with 5X sample buffer (Thermo Fisher Scientific, USA). The loaded proteins were transferred to a nitrocellulose membrane (Amersham, USA) using an electrophoretic transfer system (BioRad, USA) at 100 V for 1.5 h. The membranes were washed with a Tris-buffered saline solution (pH 7.6) containing 0.05% Tween 20 (TBST) and then blocked in TBST containing 5% skim milk (Santa Cruz Biotechnology, USA) for 1.5 h at room temperature. The membranes were then incubated at 4 °C overnight with mouse monoclonal anti-PrP antibody SAF84 (1:200; Cayman, USA) [22]. After washing in TBST, the membranes were incubated with horseradish peroxidase-conjugated secondary antibodies (Sigma-Aldrich, USA) for 1 h and washed in TBST again. The target protein bands were visualized by autoradiography using a Pierce ECL kit (Thermo Fisher Scientific, USA).

### Lipid extraction

Lipid extracts were prepared by the modified Bligh and Dyer method as described in a previous study [23]. In brief, each sample was treated with 3 mL of solution (2:1, MeOH: chloroform, v/v) and then shaken and incubated for 20 min. One milliliter of chloroform and 1.8 mL of water were added to the sample and vortexed for 30 s. The lower layer was obtained by centrifugation (1750 *g*) for 10 min at 10 °C, transferred to a new tube and dried under N_2_. Finally, all dried samples were reconstituted with MeOH/chloroform (200 μL, 1:1) and treated by ultrasound for 5 min. Then, the clear solution was used for LC/MS analysis after centrifugation (1750 *g*) for 10 min at room temperature. The recovery rates of lipid standards (SPLASH^®^ LIPIDOMIX^®^ Mass Spec Standard, Avanti polar lipids, UK) were checked to confirm the efficiency of lipid extraction for later unbiased lipid analysis, and the results showed more than 50% recovery rates.

### Lipid analysis using LC/MS

A liquid chromatograph triple-quadrupole mass spectrometer (Agilent Technologies, USA; Metabolomics Research Center for Functional Materials, Kyungsung University) with an electrospray ion source (ESI) was utilized in the present study. Chromatic separation was achieved using an XSelect CSH C18 Column (3.5 µm, 4.6 mm X 100 mm, Waters, USA). The column temperature was set at 55 °C. A binary mobile phase system was described as follows: mobile phase A: acetonitrile/water (60:40) with 10 mM ammonium formate and 0.1% formic acid; and mobile phase B: Isopropanol/acetonitrile (90:10) with 10 mM ammonium formate and 0.1% formic acid. The mobile phase constitution was changed as follows: initiation at 40% B followed by a linear gradient to 43% B over 2 min, 50% B at 2.1 min, 54% B at 12 min, 70% B at 12.1 min, 99% B at 18 min, and 40% B at 18.1 min for 1.9 min. The flow rate was set at 400 µL/min. The injection volume was 20 μL. The mass spectrometry analysis was performed using ESI in negative and positive modes. The capillary voltages were set under the negative and positive modes as follows: 2.0 kV (for positive) and 1.0 kV (for negative).

### Data processing

Mass Profiler Professional software (Agilent Technologies, USA) was employed to visualize, process and interpret multidimensional LC/MS data. Data were further normalized using total ion intensity. Analyses were performed based on multivariate statistical methods, including principal component analysis. The peak height intensity of the differential lipid metabolites was compared by a two-way analysis of variance (ANOVA) using statistical software to confirm the biomarker alterations between non-infected mice and prion-infected mice. Differentially up- or downregulated lipids between these two groups were defined as changes in lipids with values of |Log_2_ FC|> 1 and *P* < 0.05.

### Pathway analysis

To carry out the pathway analysis, a LIPEA [24] based on a database source including Kyoto Encyclopedia of Genes and Genomes (KEGG) was used to visualize relevant pathways of potential lipid biomarkers.

## Results

### Detection of PrP^Sc^

**Figure 1:**
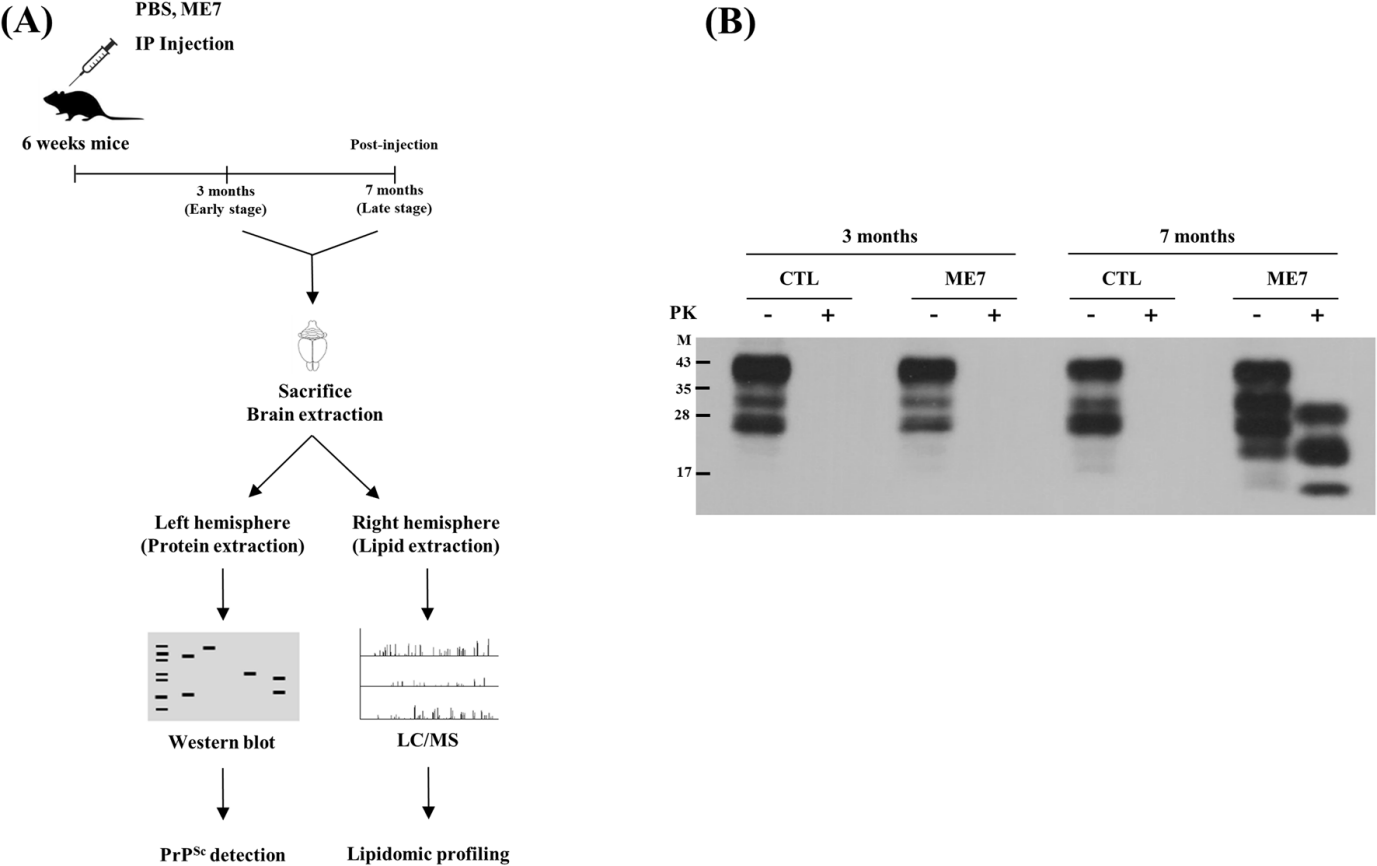
**Lipidomic profiling from prion-infected mice.** **A** Overall workflow for PrP^Sc^ detection and lipidomic profiling from prion-infected mice. IP: intraperitoneal; LC/MS: liquid chromatography mass spectrometry **B** Western blotting detection of PrP^Sc^ in the brain. The prion protein (PrP) was detected with mouse monoclonal anti-PrP antibody SAF84 (1:200; Cayman, USA). Lane 1: Proteinase K-untreated whole brain from C57BL/6 mice inoculated with PBS (CTL) at 3 months post-injection. Lane 2: Proteinase K-treated whole brain from C57BL/6 mice inoculated with PBS at 3 months post-injection. Lane 3: Proteinase K-untreated whole brain from C57BL/6 mice inoculated with ME7 scrapie (ME7) at 3 months post-injection. Lane 4: Proteinase K-treated whole brain from C57BL/6 mice inoculated with ME7 scrapie at 3 months post-injection. Lane 5: Proteinase K-untreated whole brain from C57BL/6 mice inoculated with PBS at 7 months post-injection. Lane 6: Proteinase K-treated whole brain from C57BL/6 mice inoculated with PBS at 7 months post-injection. Lane 7: Proteinase K-untreated whole brain from C57BL/6 mice inoculated with ME7 scrapie at 7 months post-injection. Lane 8: Proteinase K-treated whole brain from C57BL/6 mice inoculated with ME7 scrapie at 7 months post-injection. Bars on the left indicate molecular size markers (kDa). -: proteinase K-untreated lanes; +: proteinase K-treated lanes.

Detailed information on the study workflow is described in Figure 1A. We inoculated the ME7 mouse scrapie strain by intraperitoneal route to mice because of a high success rate of infection, low mortality rate and small inter-individual differences compared to intracranial injection. To detect PrP^Sc^ in the brains of non-infected mice and prion-infected mice, we carried out a western blot analysis coupled with a proteinase K digestion assay (Figure 1B). At 3 months post-injection (early stage), PrP^Sc^ was not detected in both non-infected mice and prion-infected mice (Figure 1B, lanes 2, 4). At 7 months post-injection (disease end stage), as expected, the PrP^Sc^ band was not detected in the non-infected mice but was detected in the prion-infected mice (Figure 1B, lanes 6 and 8).

### Principal component analysis

**Figure 2:**
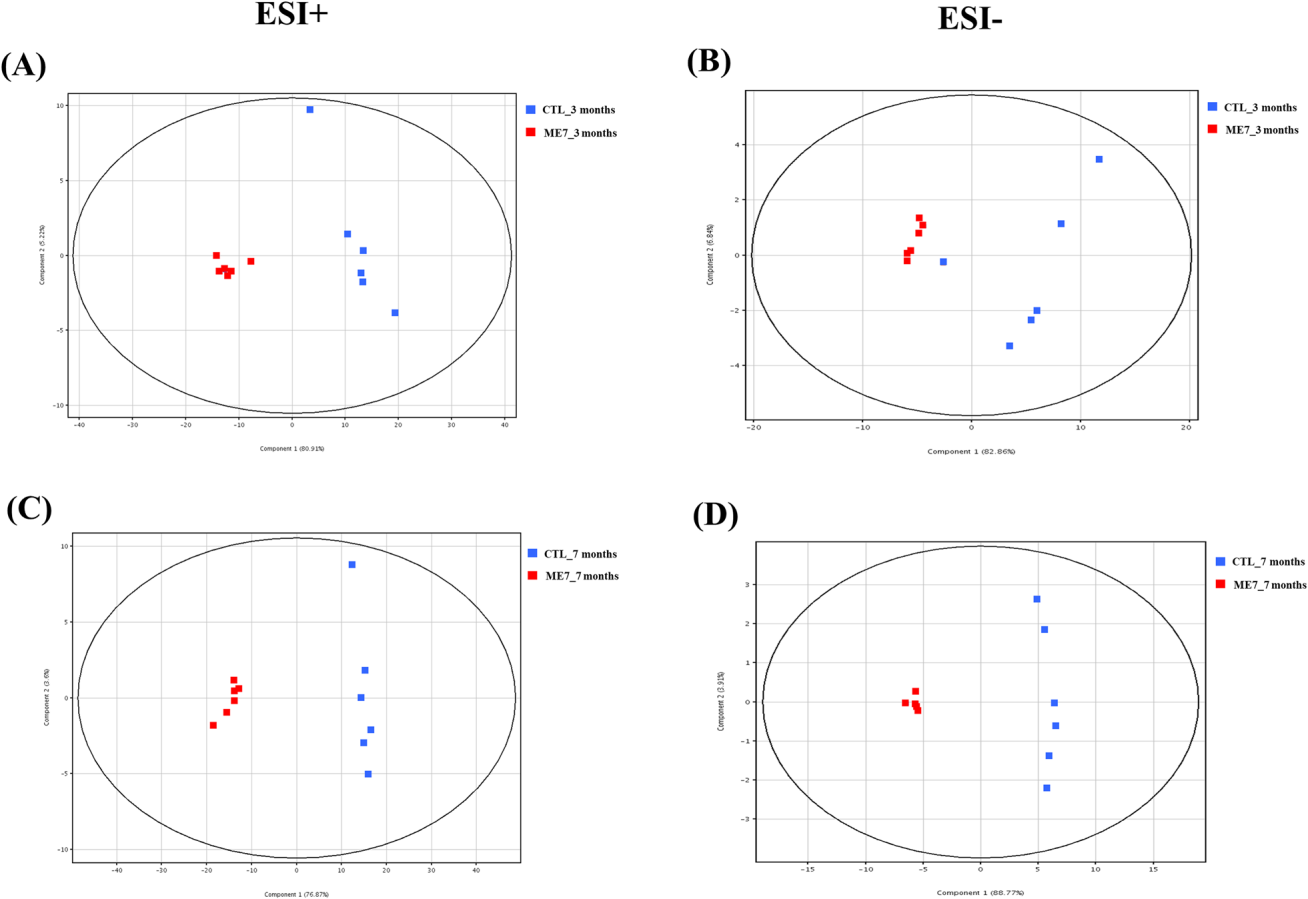
**Principal component analysis (PCA) plots of the statistical model for lipidomic data from brain samples in the negative and positive ion modes of HPLC-QTOF/MS. A** PCA plot for brain samples at 3 months post-injection in positive ion mode (ESI +). **B** PCA plot for brain samples at 3 months post-injection in negative ion mode (ESI-). **C** PCA plot for brain samples at 7 months post-injection in the positive ion mode. **D** PCA plot for brain samples at 7 months post-injection in the negative ion mode. Blue dots indicate lipidomic data for the brains of C57BL/6 mice inoculated with PBS (CTL). Red dots indicate lipidomic data for the brains of C57BL/6 mice inoculated with ME7 scrapie (ME7).

To compare the lipidomic data response to prion infection at early and late stage, a principal component analysis was performed using total Log_2_FC datasets without threshold restrictions. Although higher dispersion was observed in non-infected mice than in prion-infected mice, each principal component analysis showed well-aligned clusters of the lipidomic data at 3 and 7 months post-infection in prion-infected mice (Figure 2).

### Potential lipid biomarkers of prion disease

**Figure 3:**
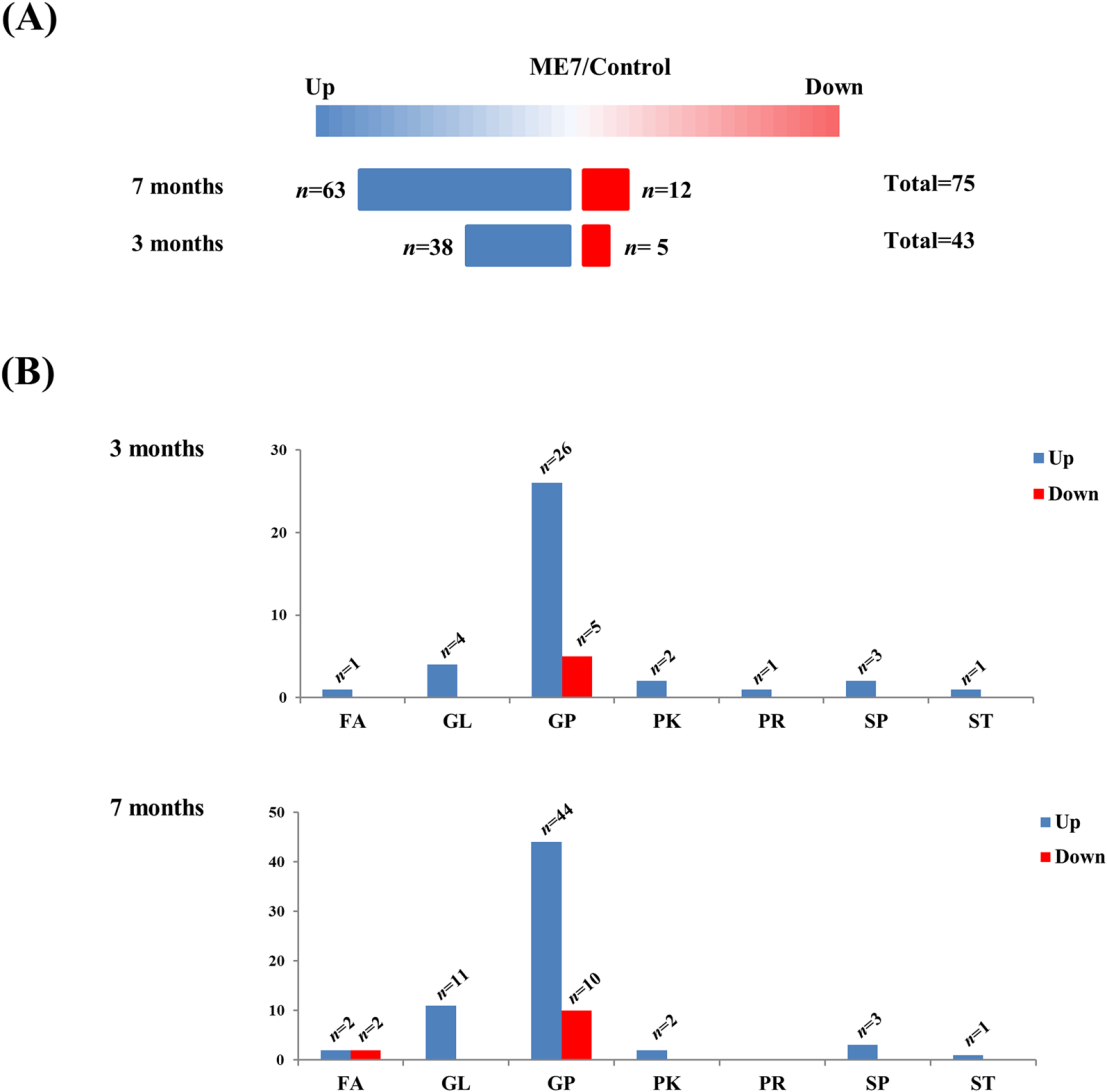
**Summary of the lipidomic profiling in prion-infected mice.** **A** Number of lipids showing the level of relative increase and decrease (|Log_2_ FC|> 1, *P* < 0.05) in prion-infected mice at 3 and 7 months post-infection. **B** Number of lipids showing the level of relative increase and decrease (|Log_2_ FC|> 1, *P* < 0.05) according to the lipid class. FA: fatty acids; GL: glycerolipids; GP: glycerophospholipids; PK: polyketides; PR: prenol lipids; SP: sphingolipids; ST: sterol lipids.

**Figure 4:**
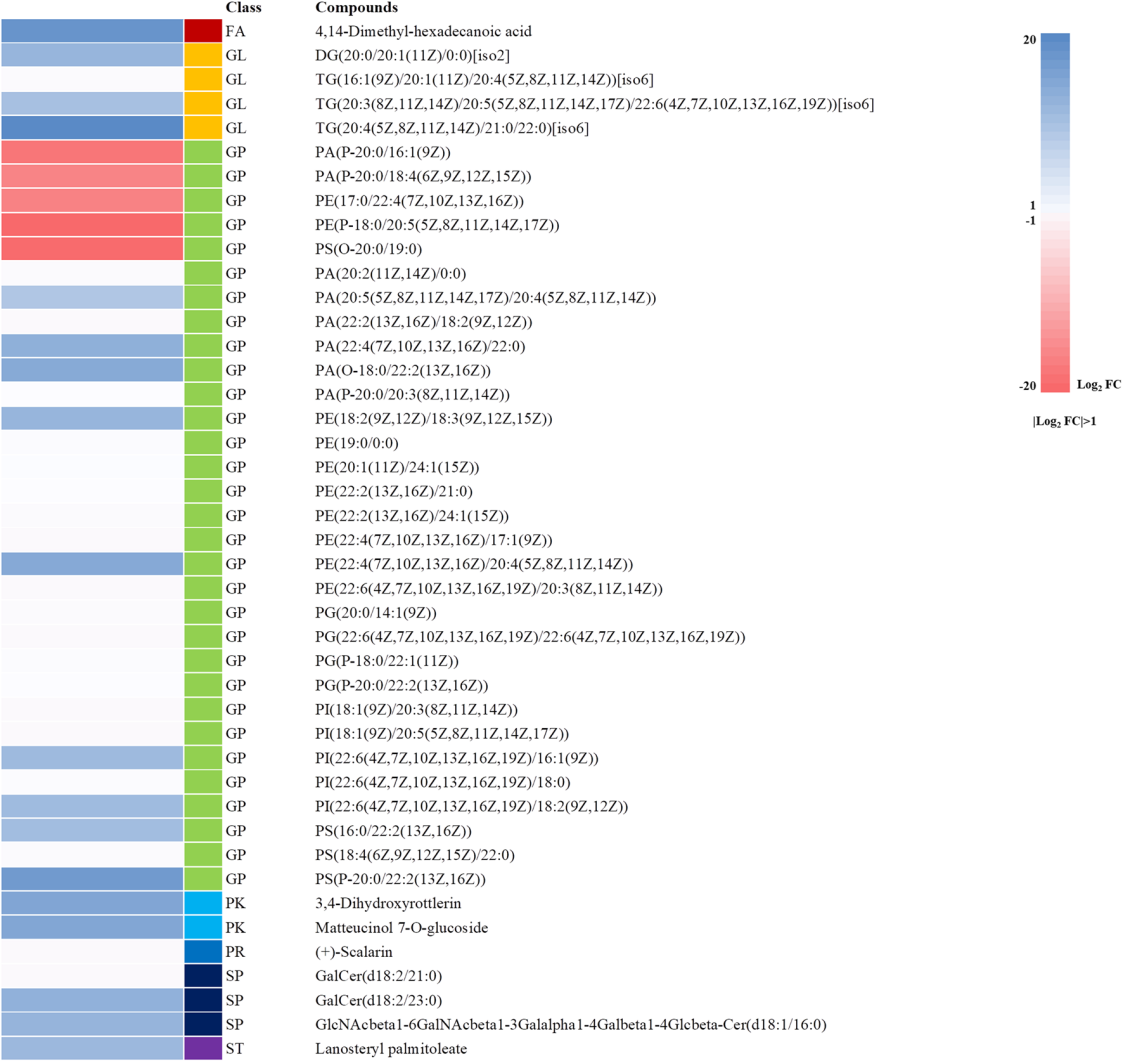
**Alteration of lipids in prion-infected mice at 3 months post-infection.** Heatmap visualized for the fold change (|Log_2_ FC|> 1, *P* < 0.05) of all potential lipid biomarkers in the brain of prion-infected mice at 3 months post-infection, showing the level of relative increase (blue) and decrease (red). FA: fatty acids; GL: glycerolipids; GP: glycerophospholipids; PK: polyketides; PR: prenol lipids; SP: sphingolipids; ST: sterol lipids.

**Figure 5:**
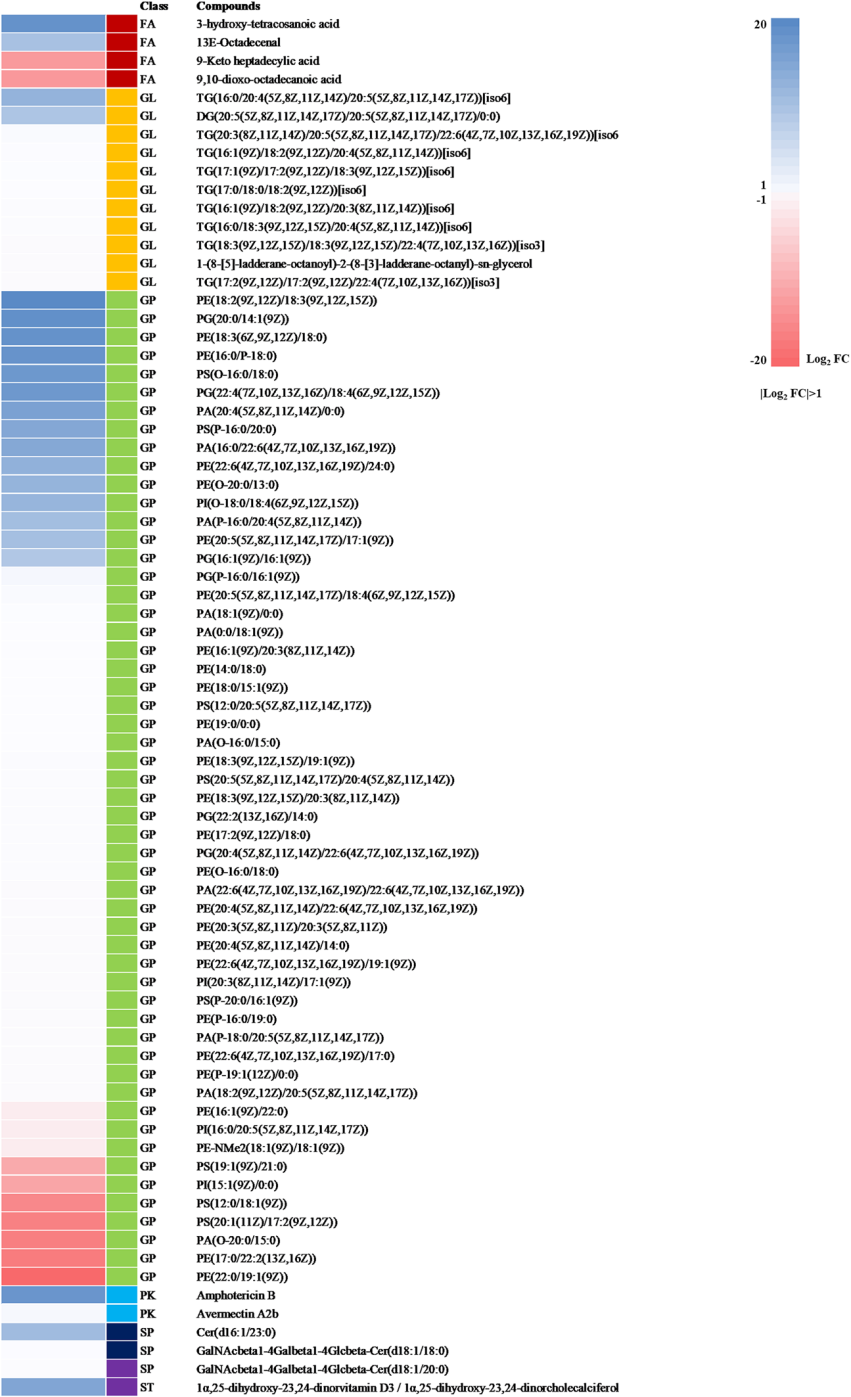
**Alteration of lipids in prion-infected mice at 7 months post-infection.** Heatmap visualized for the fold change (|Log_2_ FC|> 1, *P* < 0.05) of all potential lipid biomarkers in the brain of prion-infected mice at 7 months post-infection, showing the level of relative increase (blue) and decrease (red). FA: fatty acids; GL: glycerolipids; GP: glycerophospholipids; PK: polyketides; PR: prenol lipids; SP: sphingolipids; ST: sterol lipids.

Potential lipid biomarkers in the brains of prion-infected mice at 3 and 7 months post-infection were further extracted based on multivariate statistical analyses, with the parameters of the altered lipids satisfying the criteria |Log_2_ FC|> 1 and *P* < 0.05. In brief, a total of 43 and 75 compounds showed alterations at 3 and 7 months after ME7 inoculation, respectively (Figure 3A). In the mouse brain at 7 months post-injection, 63 and 12 compounds were upregulated and downregulated, respectively. In the mouse brain at 3 months post-injection, 38 and 5 compounds were upregulated and downregulated, respectively. We classified altered lipids according to their lipid classes (Figure 3B). A total of 7 lipid classes, i.e., fatty acids, glycerophospholipids, polyketides, prenol lipids, sphingolipids, and sterol lipids, were identified at 3 months post-infection, whereas 6 lipid classes (excluding prenol lipids) were identified in the mouse brain at 7 months post-injection. Notably, approximately 75% of the total lipid alterations in our analysis consisted of glycerophospholipids at 3 and 7 months post-infection. The alteration of lipids at 3 and 7 months post-infection was visualized by heatmaps (Figures 4, 5). Detailed information on the alteration of lipids is described in Additional files 1, 2. Notably, 4 lipids including PG [20:0/14:1 (9Z)], PE (19:0/0:0), PE [18:2 (9Z, 12Z)/18:3 (9Z, 12Z, 15Z)] and TG [20:3 (8Z, 11Z, 14Z)/20:5 (5Z, 8Z, 11Z, 14Z, 17Z)/22:6 (4Z, 7Z, 10Z, 13Z, 16Z, 19Z)] [iso6] were commonly upregulated in the brain of prion-infected mice at both 3 and 7 months post-infection.

### Pathway analysis

**Figure 6:**
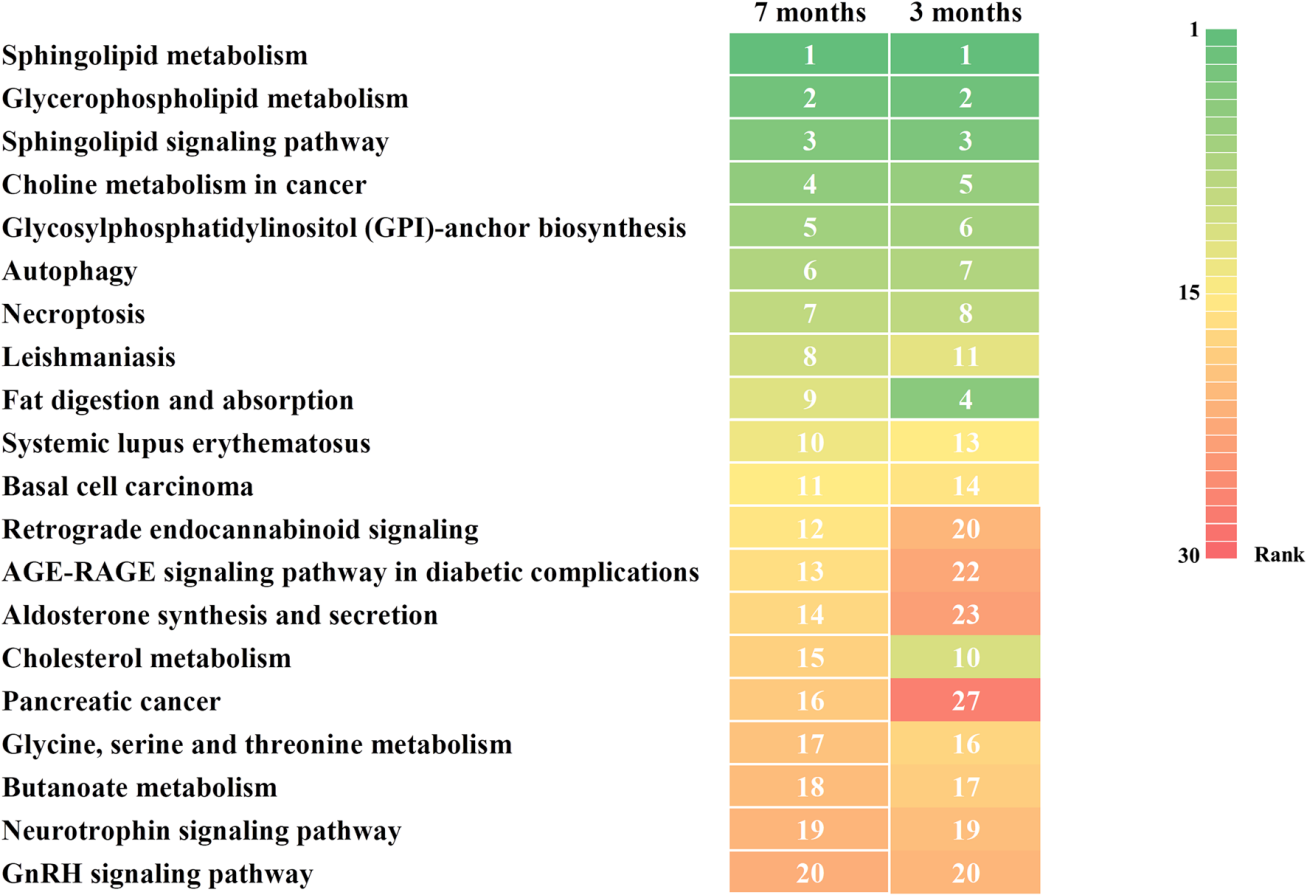
**Heatmap displaying the top 20 signaling pathways enriched at 7 months post-infection and their enrichment at 3 months post-infection.** Numbers and colors indicate the ranking (high: green; middle: yellow; low: red) of the respective signaling pathways.

To investigate the lipid alteration-related pathways, we performed a pathway analysis by LIPEA based on the KEGG database source (Figure 6). The heatmap showed the top 20 signaling pathways enriched at 7 months post-infection and their enrichment at 3 months post-infection. As expected based on the alterations in lipids, sphingolipid metabolism and signaling pathways and glycerophospholipid and choline metabolism-related pathways were commonly enhanced at both 3 and 7 months post-infection. Interestingly, the brains at 3 and 7 months post-infection from prion-infected mice showed enhancement of GPI-anchor biosynthesis, autophagy and necroptosis-related pathways.

## Discussion

In the present study, although we did not detect PrP^Sc^ in ME7 scrapie-injected mice at 3 months (Figure 1B), global lipidomic changes were found in the early stage of prion disease (Figure 4). However, since the detection limit of PrP^Sc^ by western blot analysis is relatively low with approximately 4 ng, PrP^Sc^ may not be detected in prion-infected mice at 3 months post-infection. Thus, to resolve these limitations, further investigations using a more sensitive assays including protein misfolding cyclic amplification (PMCA) and real-time quaking-induced conversion (RT-QuIC) are needed. In addition, we also identified a total of 43 and 75 novel biomarkers at 3 and 7 months, respectively (Figures 3A, 4 and 5). In a previous study, the single lipid substance phosphatidylethanolamine accelerated the conversion of PrP^C^ to PrP^Sc^; thus, the altered lipids found in this study need to be investigated to further understand whether they participate to the disease and/or the conversion processes [25]. Notably, 4 lipids including PG [20:0/14:1(9Z)], PE (19:0/0:0), PE [18:2 (9Z, 12Z)/18:3 (9Z, 12Z, 15Z)] and TG [20:3 (8Z, 11Z, 14Z)/20:5 (5Z, 8Z, 11Z, 14Z, 17Z)/22:6 (4Z, 7Z, 10Z, 13Z, 16Z, 19Z)] [iso6] were commonly upregulated in prion-infected mice at both 3 and 7 months post-infection. These lipids are worth investigating the possibility as potential biomarkers for prion disease in the future. In addition, we also found several lipid-related pathways in our animal model of prion disease (Figure 6). Among them, we found that sphingolipid metabolism and signaling pathways and the GPI-anchor biosynthesis pathway are related to lipidomic profiling. Since sphingolipids and GPI anchors are major lipid rafts components, which are prerequisites for the conversion of PrP^Sc^, this result was noteworthy [19, 20]. In a previous study, sphingolipid depletion increased the conversion of PrP^C^ into PrP^Sc^ in neuroblastoma cells [25]. In addition, several inhibitors of cellular cholesterol, the major lipid rafts component, were associated with a decrease in PrP^Sc^ formation [16–18]. Thus, the investigation of the relationship between altered levels of sphingolipids, GPI-anchor-related lipids and prion diseases is highly desirable in the future.

Although the cholesterol metabolism pathway showed a relatively low rank in prion-infected mice, the acceleration effect of cholesterol in PrP^Sc^ formation was evident in previous studies [26, 27]. In addition, cholesterol metabolism was changed in prion-infected neuronal cells and GT-1 cells and cholesterol biosynthesis was upregulated in primary hippocampal neurons. Furthermore, cholesterol accumulated in 22L scrapie-infected N2a cells and the mouse brain [26, 27]. We also found that 2 lipids in sterol class including lanosteryl palmitoleate and 1α,25-dihydroxy-23,24-dinorvitamin D3/1α,25-dihydroxy-23,24-dinorcholecalciferol were upregulated in ME7 scrapie -infected mice at 3 and 7 months post-infection, respectively. These results indicate that our findings correlated with previous studies despite the use of different prion strains. Thus, further investigation of the association between prion diseases and cholesterol metabolism pathway-related altered lipids identified in this study is warranted.

The exact mechanism of lipid alteration in cell/organelle membranes in prion diseases remains elusive, however, previous studies suggested that the conversion of PrP^C^ into PrP^Sc^ occurs on lipid rafts and PrP^Sc^-induced signal affects cell viability via the lipid-mediated cellular signal pathway [28, 29]. In addition, neuronal loss, astrocytosis and activated-microglia are predefined prion symptoms and also are related to global alteration of lipids [30, 31]. Thus, cell type and cellular compartment-specific lipidomic analyses are needed in the future to improve our knowledge of prion pathogenic mechanisms.

In the present study, we also found that the autophagy pathway is related to lipidomic profiling (Figure 6). In detail, 4,14-Dimethyl-hexadecanoic and 9,10-dioxo-octadecanoic acid were interpreted to be associated with the autophagy-related pathway (data not shown). Prion diseases are caused by the accumulation of misfolded PrP, and the autophagy signaling pathway may be initially activated to eliminate PrP^Sc^. Conversely, the activation of autophagy beyond cellular capacity induces dysfunctional autophagy and may contribute to the pathophysiology of the disease [32, 33]. Our findings indicate that both the early and late stages of the disease pathogenesis are commonly related to autophagy-related pathways at the lipid level. In addition, the necroptosis pathway has also been shown to be related to lipidomic profiling. In a previous study, a cell culture model of prion disease showed elevated biomarkers of necroptosis. Our findings provide supporting data that prion diseases are also involved in necroptosis at the lipid level.

In a recent study, a lipidomic analysis of a mouse model of Alzheimer’s disease showed that it is strongly associated with sphingolipid metabolism, glycerophospholipid metabolism and arachidonic acid metabolism pathways [34]. Interestingly, our mouse model of prion disease displayed increased sphingolipid metabolism and glycerophospholipid metabolism, which is consistent with that of Alzheimer’s disease; however, the arachidonic acid metabolism pathways did not show any increase in the brain of prion-infected mice at 3 months post-infection, and these pathways showed a very low rank in prion-infected mice at 7 months post-infection (data not shown). Conversely, our mouse model of prion disease presented unique pathways compared to those of Alzheimer’s disease, including choline metabolism and GPI-anchor biosynthesis (Figure 6). Prion diseases and Alzheimer’s disease are neurodegenerative diseases caused by misfolded proteins, PrP^Sc^ and amyloid beta, and they show similar pathophysiological features, including transmissibility, endoplasmic reticulum stress and autophagy-related responses [32, 35, 36]. In addition, 13% of clinically diagnosed Alzheimer’s disease patients were rediagnosed as having human prion disease post-mortem [37]. These studies indicate that there is difficulty in distinguishing between prion diseases and Alzheimer’s disease and that there is a need for a clear understanding of the differences between these two diseases. Thus, the unique lipidomic mechanism identified in the present study may be characteristic to prion disease. In addition, 4 lipids including PG [20:0/14:1 (9Z)], PE (19:0/0:0), PE [18:2 (9Z, 12Z)/18:3 (9Z, 12Z, 15Z)] and TG [20:3 (8Z, 11Z, 14Z)/20:5 (5Z, 8Z, 11Z, 14Z, 17Z)/22:6 (4Z, 7Z, 10Z, 13Z, 16Z, 19Z)] [iso6] were commonly upregulated in prion-infected mice at both 3 and 7 months post-infection. Since these lipids were not altered in Alzheimer’s disease and specifically altered in the prion-infected animal model, these lipids may be considered as potential specific biomarkers of prion diseases. However, since the present study was performed using only one prion strain (ME7), these results could be prion strain-specific. Further validation with other prion strains including 22L, RML and 139A is highly desirable in the future.

In the present study, we first carried out a large-scale lipidomic profiling in an animal model of prion disease and identified a total of 43 and 75 novel potential biomarkers at 3 and 7 months post-infection, respectively. Notably, approximately 75% of the total lipid alterations at 3 and 7 months post-infection were accounted for by glycerophospholipids. In addition, altered lipids between non-infected and prion-infected mice are related to lipid raft-related pathways. In the present study, we found novel potential biomarkers and therapeutic targets of prion disease. To the best of our knowledge, this was the first large-scale lipidomic profiling of prion disease.

## Supplementary Information

**Additional file 1.** **Comparison of lipid between non-infected and prion-infected mice at 3 months post-infection.**

**Additional file 2.** **Comparison of lipid between non-infected and prion-infected mice at 7 months post-infection.**

## Abbreviations

PrP: Prion protein
PrP^Sc^: Abnormal prion protein
PrP^C^: Normal prion protein
LC/MS: Liquid chromatography mass spectrometry
GPI: Glycosylphosphatidylinositol
