# Supplementary material for: Large-scale lipidomic profiling identifies novel potential biomarkers for prion diseases and highlights lipid raft-related pathways
Source: Vet Res. 2021 Jul 21;52:105. doi: 10.1186/s13567-021-00975-1 (PMC8296529; doi:10.1186/s13567-021-00975-1)
Supplement: Supplementary file 2 — Additional file 2. Comparison of lipid between non-infected and prion-infected mice at 7 months post-infection. [file 13567_2021_975_MOESM2_ESM.docx]

**Additional file 2** **Comparison of lipid between wild type and prion disease infected mice at 7 months**
